# Extraction of Chlorogenic Acid Using Single and Mixed Solvents

**DOI:** 10.3390/molecules30030481

**Published:** 2025-01-22

**Authors:** Hyeon Ji Jeon, Bong-Seop Lee, Changhyup Park

**Affiliations:** 1 Department of Chemical Engineering, Kangwon National University, Chuncheon 24341, Gangwon, Republic of Korea; junhgii@kangwon.ac.kr; 2 Department of Integrative Engineering for Hydrogen Safety, Kangwon National University, Chuncheon 24341, Gangwon, Republic of Korea

**Keywords:** chlorogenic acid, COSMO-SAC, Hansen solubility parameter, extraction, mixed solvents

## Abstract

Chlorogenic acid, which is extracted from a wide range of natural sources, is attracting the attention of many researchers in the pharmaceutical and biomedical fields due to its various positive effects, such as such as anti-inflammatory and antibacterial properties. Considering the effects of economics and solvent toxicity, water, ethanol, and their mixtures were selected as the solvents for extracting chlorogenic acid at various temperatures (298~318 K) and over a whole range of concentrations. The solubility of chlorogenic acid increased with temperature regardless of the solvents, and the solubility was higher in pure ethanol than in pure water. The solubility of chlorogenic acid in mixed solvents exhibited a gradual rise as the water content increased, reaching a maximum at a specific water weight fraction. These trends were well predicted by the COSMO-SAC model and Hansen solubility parameter method. By comparing the σ-profile, it was confirmed that the maximum solubility in mixed solvent comes from the similarity of σ-profiles between chlorogenic acid and mixed solvent which represents the surface charge density of the molecule.

## 1. Introduction

Chlorogenic acid, a depside acid formed by the esterification of quinic acid (1-hydroxyhexahydrogallic acid) and caffeic acid, is a phenolic compound that was first isolated from green coffee beans, which are unroasted coffee beans [1]. In addition to green coffee beans, chlorogenic acid, which is extracted from a wide range of natural sources such as strawberries [2], blueberries [3], folium eucommiae [4], and potatoes [5], is garnering significant interest from many researchers in the pharmaceutical and biomedical fields due to its various positive effects, such as anti-inflammatory [6,7,8,9,10,11] and antibacterial [12,13].

Lemos et al. [6] measured the contents of metabolites, caffeine, and chlorogenic acid in 33 coffee bean samples using a UV-vis spectrophotometer. To evaluate the high antioxidant activity of chlorogenic acids, they used the Ferric Reducing Ability of Plasma (FRAP) method, which measures the ferric reducing ability in plasma [14], the ABTS assays method, which gauges antioxidant properties by measuring the ability to quench chromogenic radical cation ABTS (2,2-azinobis (3-ethyl-benzothiazoline-6-sulphonate) radical cation [15], and the DPPH (2,2-diphenyl1-picrylhydrazyl) method, which measures antioxidant properties by observing the color change resulting from neutralizing the DPPH free radical with antioxidant substances [16]. Moreira et al. [7] also used high-performance liquid chromatography (HPLC) to measure the contents of chlorogenic acids and caffeine in coffee beverage samples according to the degree of roasting. They then confirmed, using the FRAP method, that chlorogenic acids, not caffeine, have antioxidant capacity. Thom [17] and Shimoda et al. [18] concluded that the consumption of coffee rich in chlorogenic acids can prevent weight gain and fat accumulation, as they obtained results showing that weight decreased when consuming coffee rich in chlorogenic acid continuously. Watanabe et al. [19] explained that chlorogenic acid has the effect of lowering blood pressure when consumed. Bassoli et al. [20] examined the impact of chlorogenic acid on hepatic glucose output, glucose tolerance, and blood glucose levels. Their findings revealed that chlorogenic acid lowered the plasma glucose peak during an oral glucose tolerance test, suggesting its potential as an agent for reducing the glycemic index. In addition, studies have been published that show that chlorogenic acids have protective effects on the liver and kidneys [21,22], and anti-inflammatory [23,24] and antibacterial properties [12,13].

Various studies have been conducted to efficiently extract chlorogenic acid from various extraction sources. Budryn et al. [25] extracted chlorogenic acids from coffee beans under various conditions, including brewed, boiled, and pressure-increased boiling, and roasted to different degrees (light, medium, and dark roasted). They compared which method and conditions achieved the highest extraction efficiency. Liu et al. [26] extracted chlorogenic acids from folium eucommiae using ultrasonic-assisted extraction (UAE) and compared it with other extraction methods, extraction times, and extraction efficiency. Azevedo et al. [27] extracted chlorogenic acids, caffeine, and coffee oil from green coffee beans using pure supercritical fluid carbon dioxide (SCF CO_2_), and extracted chlorogenic acids using a combination of co-solvents and SCF CO_2_, comparing the results from the two methods. Chen et al. [28] used microwave and ultrasound extraction to extract chlorogenic acid from a significant amount of tobacco residue, a waste product in cigarette manufacturing, using a mixed solvent of acetone and water. They analyzed the extracted substance and found, through HPLC, that the selectivity of chlorogenic acid was excellent among the constituents of the extracted material. In order to achieve high extraction efficiency of chlorogenic acid by these various methods, data research on the solubility of chlorogenic acid under various conditions, such as solvent and temperature, is needed.

Chlorogenic acid is a compound that has been shown to have a number of health benefits. However, it is not water-soluble, which makes it difficult to study and measure its effects. In order to overcome this challenge, some researchers have used ethanol/water mixtures as solvents. For this reason, in several studies [26,29,30,31,32,33,34], a solvent mixture of ethanol and water has been used to increase solubility and measure various solubilities, such as chlorogenic acid. For example, Bowden et al. [29] measured the solubility of proteinogenic α-amino acids in ethanol/water mixed solvents (0, 25, 50, 75, 100 wt% of ethanol) at 0.1 MPa pressure and 298.15 K. Lourdes, et al. [26] determined the solubility of D-glucose in ethanol/water mixed solvents (40, 60, 70, 80, 100 wt.% of ethanol) in the temperature range of 283 K to 333 K. Cuevas-Valenzuela et al. [34], employing a novel shake-flask method coupled with UV spectrophotometry, measured the solubility of (+)-catechin in ethanol/water mixed solvents (~13.0 wt.% of ethanol) across the temperature range of 277.6 K to 331.2 K at atmospheric pressure. Both experiments demonstrated an increase in solubility with temperature elevation and variations in solubility based on the proportion of ethanol. These diverse solubility measurements in various temperatures and ethanol/water mixed solvents could aid in optimizing extraction process conditions for respective substances. However, there are not enough solubility data for chlorogenic acid in water/ethanol mixtures.

In general, in order to model the phase equilibrium at constant temperature and pressure using activity coefficient models and equations of state, pure component parameters and binary interaction parameters are estimated by regressing saturation vapor pressure and density data. For biomolecules, often vapor pressure data are unavailable due to their low thermal stability. Moreover, a substantial amount of experimental data is required to estimate relatively accurate pure and binary interaction parameters. The process of estimating these parameters demands considerable time and validation of the estimated parameters. However, COSMO-based models based on computational chemistry (COSMO-RS [35,36] and COSMO-SAC [37,38]) are suitable for predicting solubility in appropriate pure and mixed solvents as they do not require pure component and binary interaction parameters. Therefore, in this study, due to these reasons, solubility was predicted using the COSMO-SAC model, which is free from thermodynamic consistency issues [37] among COSMO-based models and is provided as an open-source solution [39].

Direct experimentation to select extraction conditions for high extraction efficiency is accurate, but it requires considerable time, cost, and effort. Therefore, there is research on predicting solubility using predictive thermodynamic models. The validity of prediction results with the COSMO-SAC model for the solubility of biomolecules in pure and mixed solvents has been verified by many research groups [38,40,41,42,43,44,45,46]. Lee and Lin [38] investigated the solubility of paracetamol in 2624 single ionic liquids and in 3,441,376 two ionic liquids mixtures using the COSMO-SAC model [47]. Kang et al. [48] predicted and compared the solubility of carboxylic acids using the COSMO-SAC [49] model and the UNIFAC [50,51] model. Tung et al. [40] predicted the solubility of pharmaceuticals using the nonrandom two-liquid segment activity coefficient (NRTL-SAC) [52] and the COSMO-SAC [37] model. The Hansen solubility parameter estimation method [53,54,55], which utilizes group contribution, is also used to investigate the influence of solvents on solute solubility. For instance, Shekaari et al. [55] employed the Hansen solubility parameter estimation method to select the optimal extraction solvent for the hesperidin drug in aqueous, biodegradable, acidic deep eutectic solvents based on choline chloride. Although these predicted data are still limited and not accurate, they can be a good reference for selecting extraction conditions through the prediction of experimental data.

Chlorogenic acid is gaining attention from researchers in pharmaceutical and biomedical fields due to its positive biological effects, such as anti-inflammatory [6,7,8,9,10,11] and antibacterial [12,13] properties. To utilize chlorogenic acid in these areas, an extraction process in a non-toxic solvent is required. In this study, various factors, such as the extraction temperature, type of solvent, and solvent mixing ratio, were investigated to understand the solubility behavior. The solubility of chlorogenic acid in pure water and ethanol, and their mixed solvent was measured at various temperature conditions (298.15~318.15 K) and over a whole range of concentrations (0~1 mole fraction) to provide a suitable composition of solvents and extraction temperature. The COSMO-SAC [47] model and Hansen solubility parameter method were employed to investigate the optimal extraction conditions and gain insights into the solubility behavior with respect to temperature, solvent types, and solvent composition.

## 2. Results and Discussions

To quantify the solubility of chlorogenic acid in each solvent, a calibration curve was constructed by measuring the maximum absorption wavelength of a chlorogenic acid sample with known concentration. Each sample was diluted with *DI* water considering the absorbance detection limit of UV-vis. The calibration curve was generated at a maximum absorption wavelength of 324 nm according to the concentration (ppm, mg/kg) data and the calibration curve, showing a good linearity with the coefficient of determination, R^2^ of 0.99999.

**Figure 1:**
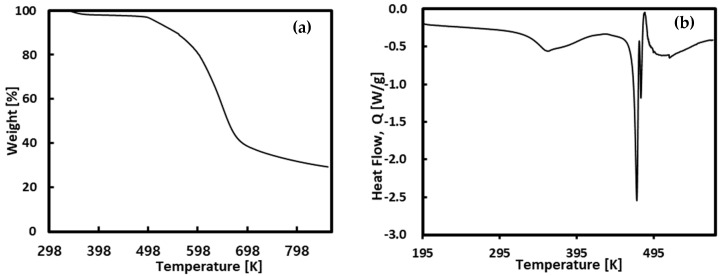
Thermal analysis (**a**) TGA and (**b**) DSC measurement of chlorogenic acid.

Chlorogenic acid has a melting point of approximately 464.15–481.15 K [56,57], but the melting enthalpy value is not well known. Therefore, DSC measurements were performed to directly measure the melting point and melting enthalpy and the standard uncertainty was estimated by the guidelines of NIST [58]. The results are reported in Table 1 with literature values and the analysis results are shown in Figure 1. Based on the TGA results, the measurement temperature range was determined to be 298~573 K to prevent contamination of the DSC measurement cell. The melting point of the chlorogenic acid was determined by detecting the onset at the beginning of the decreasing peak, and the melting enthalpy was determined by integrating the endothermic peak. Typically, the enthalpy of fusion using DSC shows an uncertainty of 3–5% [59].

**Figure 2:**
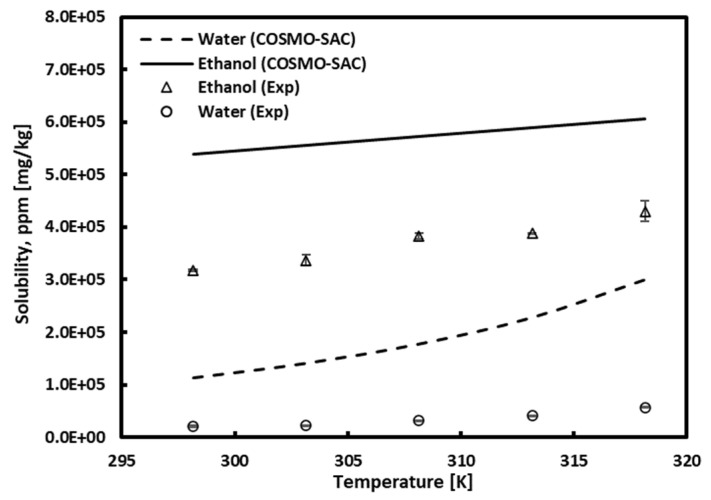
Effect of temperature on chlorogenic acid solubility in pure water and ethanol.

The solubility of chlorogenic acid in pure ethanol and water was measured at temperatures of 298.15 K, 303.15 K, 308.15 K, 313.15 K, and 318.15 K. Figure 2 depicts the solubility of chlorogenic acid in pure ethanol and water at different temperatures and atmospheric pressure (101.325 kPa), and the numerical values are reported in Table 2. At this time, the standard uncertainty of pressure is ±0.07 kPa, and the effect of pressure on solubility is negligible. Chlorogenic acid was more soluble in pure ethanol than in pure water, and the solubility increased with temperature in both cases. The COSMO-SAC model predicts higher solubilities than experimental data, but the trend of increasing solubility with temperature is consistent. The increase in solubility with temperature showed a greater tendency in ethanol according to experimental data, while predictions using the COSMO-SAC model indicated a greater tendency in water. This discrepancy in results seems more linked to the formula used to calculate solubility (see Equation (6)) rather than errors caused by the COSMO-SAC model. As confirmed in the calculation equation of solubility trends with temperature, they are determined by the melting enthalpy and melting point obtained from DSC measurements. Other literature only presented melting temperatures without providing melting enthalpy values, making comparisons challenging. However, the melting temperatures proposed by Ramesh et al. [56] show significant differences from both our presented values and those presented by Zhao et al. [57]. These variations suggest a differing temperature dependency in solubility, anticipated to result in varied predictions. Therefore, when comparing predicted results from the COSMO-SAC model with experimental data, one must consider this theoretical background.

**Figure 3:**
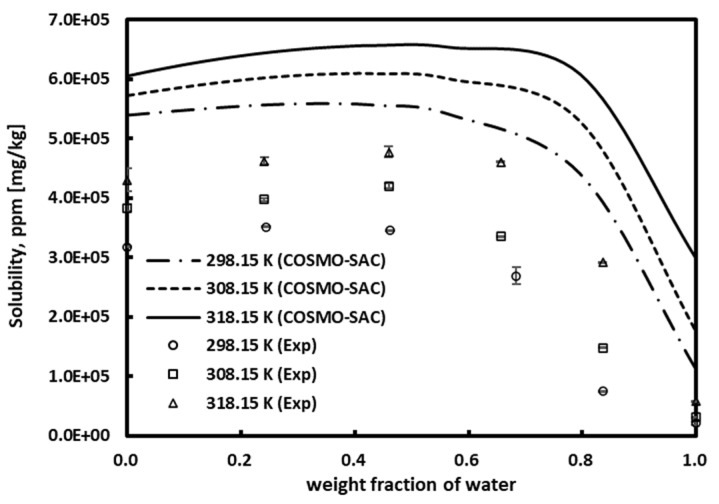
Effect of temperature on chlorogenic acid solubility in mixed H_2_O/EOH solutions.

The solubility of chlorogenic acid in ethanol/water mixtures was measured at three temperatures of 298.15 K, 308.15 K, and 318.15 K. The solubility data of chlorogenic acid at each temperature are reported in Table 3. As can be seen in Figure 3, when expressed as concentration (ppm), the solubility of chlorogenic acid exhibited a gradual rise as the water content increased, reaching a maximum at a specific water weight fraction. The solubility of chlorogenic acid then decreased sharply as the water weight fraction increased further. The solubility of chlorogenic acid increased with temperature at all temperatures. As the temperature increases, the water content of the solvent with the maximum solubility value gradually increases (see Table 3). This phenomenon is also accurately described by the COSMO-SAC model. However, it was not possible to accurately predict the composition of the water with the maximum solubility.

Overall, the COSMO-SAC model provided good predictions of the solubility trends of chlorogenic acid. Specifically, it accurately captured the trend of increasing solubility with rising temperature and the observation that solubility reaches its maximum at a specific ratio of water and ethanol in the mixed solvent. This is because the COSMO-SAC model calculates the charge density on molecular surfaces through computational quantum chemistry and describes the interactions between them. As a result, it is expected to effectively represent the interactions between chlorogenic acid and solvent molecules. This is because the COSMO-SAC model calculates the charge density on molecular surfaces using computational quantum chemistry and describes the interactions between these surfaces. Therefore, it is considered to effectively capture the interactions between chlorogenic acid and solvent molecules. However, the COSMO-SAC model tends to overestimate solubility. This is likely because chlorogenic acid contains multiple hydroxyl groups (-OH) and a carboxyl group (-COOH), which can form hydrogen bonds, leading to strong interactions with solvents with functional groups for hydrogen bonding. The COSMO-SAC model is not very accurate in describing such hydrogen bond interactions, and for this reason, continuous development is taking place [47,60,61].

Hansen solubility parameters (HSP) can also be used to select good solvents for extracting chlorogenic acid. Hansen solubility parameters δ are broken into *δ_D_*, *δ_P_*, and *δ_H_* for dispersion, polar and hydrogen bonding [62]:

*δ*^2^ = *δ*_*D*_^2^ + *δ*_*P*_^2^ + *δ*_*H*_^2^ (1)

**Figure 4:**
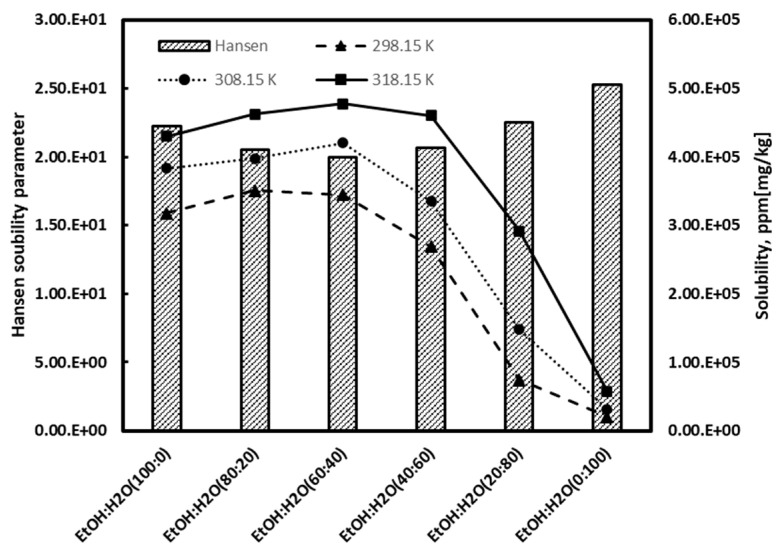
The comparison between the Hansen solubility parameter and the experimental solubility of chlorogenic acid in mixed H_2_O/EOH solvents.

The distance between the solvent (*i*) and the solute (*j*) is typically referred to as $∆\delta_{ij}$ and can be calculated using the following formula:

(2) $$∆\delta_{ij}=\sqrt{4{\left(\delta_{d}^{i}-\delta_{d}^{j}\;\right)}^{2}+{\left(\delta_{p}^{i}-\delta_{p}^{j}\right)}^{2}+{\left(\delta_{h}^{i}-\delta_{h}^{j}\;\right)}^{2}\;}$$

(3) $$\delta_{mix}=\alpha \delta_{i}+\left(1-\alpha \right)\delta_{j}$$

where α is the volume fraction of *i* in the mixed solvents of *i* and *j.* The smaller the difference in Hansen solubility parameters, the better the affinity between two substances, indicating that the solute is more likely to dissolve well in the solvent. The results obtained using the estimated Hansen solubility parameters for each component were applied to mixtures [63], and the solubility of chlorogenic acid in mixed solvents was compared in Figure 4 at 298.15, 308.15, and 318.15 K. As a result, the Hansen solubility parameters showed the following order for various EtOH:H_2_O mixed solvent ratios (60:40 < 80:20 < 40:60 < 20:80). The solvent EtOH:H_2_O 60:40 exhibited the lowest Hansen solubility parameter, while pure water, which demonstrated the lowest solubility, showed the highest Hansen solubility parameter. Although the trend of the experimental data did not perfectly align, the overall tendency matched with the previously discussed predictions of the COSMO-SAC model. Furthermore, the COSMO-SAC model is applicable for predicting the solubility of various biomolecules in a wide range of solvents, including deep eutectic solvents and ionic liquids. Such studies are widely documented in the literature [38,46,64,65]. Additionally, the COSMO-SAC model can be applied to predict the solubility of biomolecules in natural products and can be integrated with Aspen Plus for use in the design of extraction processes. Considering these points, the COSMO-SAC model is deemed more efficient and industrially applicable than the Hansen solubility parameter method.

**Figure 5:**
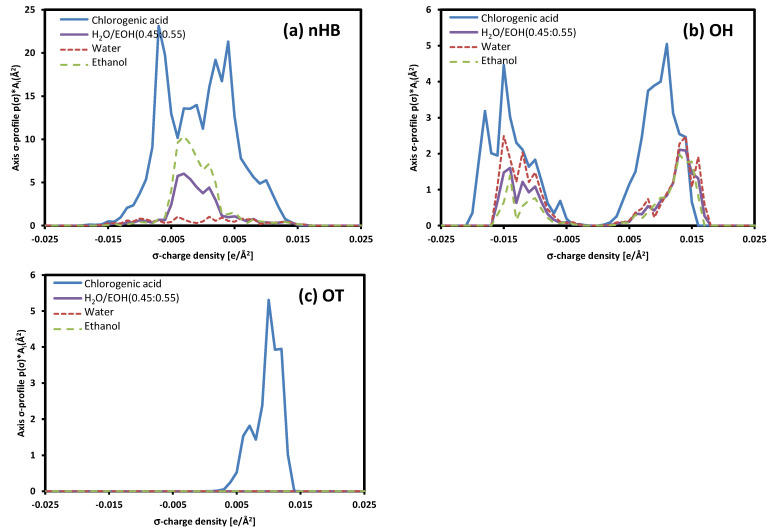
The σ-profile of each component: chlorogenic acid (bold solid line), ethanol (green dash line), water (red dotted line), and mixed-H_2_O/EOH of water and ethanol with 0.45:0.55 ratio.

The phenomenon of the maximum solubility value depending on the composition of the solvent can be clearly explained by comparing the σ-profiles. The surface of a molecule is classified into three categories: non-hydrogen bonding (nHB) surfaces, hydrogen bonding from -OH groups (OH), and any other hydrogen bonding surfaces (OT) [47]. As shown in Figure 5, the σ-profile, which represents the surface charge density of the molecule, showed a more similar trend to the σ-profile of chlorogenic acid than of each solvent in the mixture of water and ethanol. This similar σ-profile means that the surface charge density of the molecules in the mixed solvent is similar to that of chlorogenic acid, and this similarity results in increased miscibility between the mixed solvent and chlorogenic acid [38]. In addition, the significant solubility difference between pure water and ethanol can be explained by comparing the σ-profile. Around 0.0 σ-charge density area, chlorogenic acid shows a more similar trend in ethanol than in water, with a significant difference. This difference can be confirmed by the higher solubility of chlorogenic acid in ethanol.

## 3. Experiment

### 3.1. Chemicals

Chlorogenic acid was purchased from ALADDIN. The mixed solvents with ethyl alcohol and deionized water (DI water) were prepared with different molar ratios. DI water was prepared using the Direct-Q Water purification system (Millipore Corporation, Burlington, MA, USA). The purity and suppliers for used chemicals are listed in Table 4.

### 3.2. Solubility Measurement

To measure the solubility of chlorogenic acid, the absorbance was measured using a UV-vis spectrophotometer (SHIMADZU UV-2600i, Kyoto, Japan). The calibration was performed between concentration and absorbance. The solubility of chlorogenic acid in pure ethanol (EtOH) and pure water (H_2_O) was measured at atmospheric pressure and in a temperature range of 298–328 K. The solubility of chlorogenic acid in other ratios of H_2_O/EtOH solution was measured in a temperature range of 298.15 K, 308.15 K, and 318.15 K, under atmospheric pressure.

**Figure 6:**
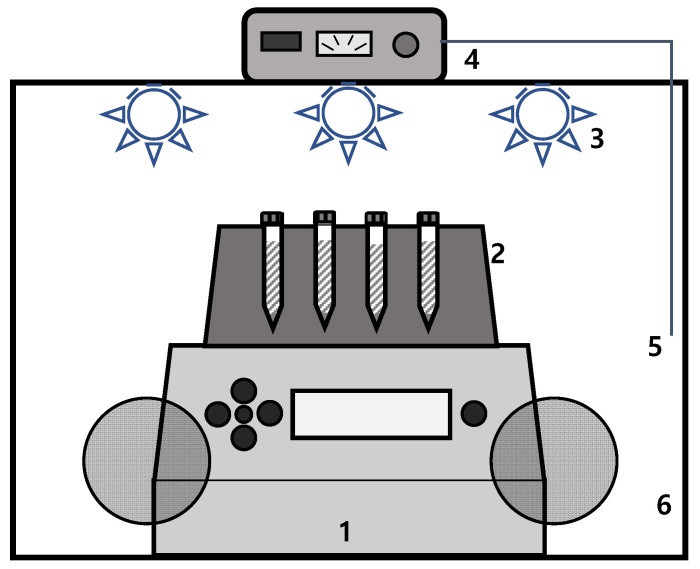
Experimental setup for solubility measurement: 1. ThermoMixer (Eppendorf, ThermoMixer C); 2. Heating block (Eppendorf, SmartBlock 15 mL); 3. Heater; 4. Temperature controller (Misung, TC200P); 5. Thermocouple; and 6. Glove box.

At a fixed temperature, a solution of supersaturated chlorogenic acid in a solvent was shaken at 600 rpm in a heating block (Eppendorf, Hamburg, Germany) for 16 h. After that, it was allowed to settle down for 6 h. The solutions with equilibrium state were extracted for solubility measurement through a preheated glass syringe equipped with a syringe filter (PTFE, pore 0.45 μm) inside the glove box. As shown in Figure 6, in order to prevent precipitation during the extraction process, the heating block was placed in a glove box, and the air temperature inside the glove box was maintained at 5 °C higher than the extraction temperature by PID temperature controller. In addition, the heated air in the glove box was circulated using a fan to obtain a uniform and constant temperature. In our preliminary experiment, when we conducted extraction experiments in the glove box without heating the airbase, we obtained significantly lower dissolution data compared to when we conducted the extraction experiment by heating the airbase. The absorbance of the extracted solutions was measured using a UV-vis spectrophotometer. The solubility was obtained through the calibration curve. The solubility data were presented as the average of at least 2 repeated experiments. The standard uncertainty is estimated by the guidelines of NIST [58].

### 3.3. Thermal Analysis

To assess the melting enthalpy and melting point, thermogravimetric analyzers (TGA, TA Instruments, New Castle, DE, USA, TGA 55) and differential scanning calorimetry (DSC, TA Instruments) with refrigerated cooling system (DSC25+RCS) were used. DSC was used to measure the melting enthalpy and melting point in the temperature range of 298 K to 573 K.

## 4. Theory and Model

The solubility of chlorogenic acid (*d*) in solution (*l*) is calculated by equating the fugacity of the chlorogenic acid in the solid and the solution phases at constant temperature *T* and pressure *P*, i.e.,

(4) $$f_{d}^{o,s}(T,P)=x_{d}\gamma_{d}(T,P,x_{i})f_{d}^{o,\ell }(T,P)$$

where *f*_*d*_^*o,s*^ and *f*_*d*_^*o,l*^ represent the fugacity of the solute in the pure solid and liquid states, respectively. The ratio of these fugacities can be determined from the melting temperature (*T_m_*) and the heat of fusion (∆*H_f_*) of the chlorogenic acid as [66]

(5) $$\ln\frac{f_{d}^{o,s}}{f_{d}^{o,\ell }}=\frac{∆H_{f}}{RT_{m}}(1-\frac{T_{m}}{T})$$

From the above two equations, the solubility of a solute in solution is obtained from

(6) $$\ln x_{d}=-\ln\gamma_{d}+\frac{∆H_{f}}{RT_{m}}(1-\frac{T_{m}}{T})$$

In this study, the ∆*H_f_* and *T_m_* of the chlorogenic acid were obtained through measurement. Then, the activity coefficient of the chlorogenic acid in the liquid phase was determined by COSMO-SAC model [47].

The COSMO-SAC model [47] characterizes the activity coefficient by accounting for interactions between molecules in contact, including both a residual (*res*) term and a combinatorial (*comb*),

(7) $$\ln{\gamma_{i}}^{COSMO-SAC}=\ln{\gamma_{i}}^{res}+\ln{\gamma_{i}}^{comb}$$

The residual term captures the non-ideality arising from variations in the attractive interactions. The Staverman—Guggenheim combinatorial term [67] describes the non-ideality due to the differences in the shape and size between molecules in the mixture. In the COSMO-SAC model, the residual term is derived from the screening charge on the molecular surface. The distribution of screening charges on the molecular surface is obtained from COSMO calculation [37,68], in which a molecule is dissolved in a perfect conductor. The electronic features of the molecule are characterized by the induced screening charges appearing on the cavity surface. To quantify this distribution, the molecular surface is divided into small segments.

The probability of finding a surface segment with a screening charge density σ*_m_*, associated with surface type *t* (*t* = *nhb*, *OH*, or *OT*), referred to as the σ-profile, is

(8) $$p_{i}^{t}\left(\sigma_{m}\right)=\frac{A_{i}^{t}\left(\sigma_{m}^{t}\right)}{A_{i}}$$

where *A_i_* is the total surface area of the species *i* and *A_i_*(σ*_m_*) = ∑_t_ *A_i_^t^*(σ*_m_^t^*) is the surface area of a segment with a screening charge density σ*_m_*. The *σ*-profile for a mixture is the mole fraction weighted sum of the *σ*-profiles of all the components, i.e.,

(9) $$p_{s}^{t}\left(\sigma_{m}^{t}\right)=\frac{\sum_{i}x_{i}A_{i}p_{i}^{t}\left(\sigma_{m}^{t}\right)}{\sum_{i}x_{i}A_{i}}$$

## 5. Conclusions

A study was conducted to measure the solubility of chlorogenic acid in pure ethanol, water, and water/ethanol mixed solutions at atmospheric pressure and 298.15~318.15 K, using UV-vis spectroscopy and modeling with the COSMO-SAC model. The solubility of chlorogenic acid increased with temperature regardless of the solvents, and the solubility was higher in pure ethanol than in pure water. The solubility of chlorogenic acid in mixed solvent exhibited a gradual rise as the water content increased, reaching a maximum at a specific water weight fraction. The COSMO-SAC calculations resulted in higher predictions of solubility compared to experimental data, showing significant discrepancies. However, the trends in solubility variation based on temperature, solvent type, and composition in mixed solvents matched the experimental data. Therefore, it is considered that the COSMO-SAC model can be sufficiently utilized as foundational data to determine the solvent, temperature, and composition in mixed solvents in order to obtain maximum solubility prior to experimentation. By comparing the σ-profile, it was confirmed that the maximum solubility in mixed solvent comes from the similarity of σ-profiles between chlorogenic acid and mixed solvent, which represents the surface charge density of the molecule.

## Figures and Tables

**Table 1:** Melting temperature and melting enthalpy of chlorogenic acid measured by DSC.

Property	This Work *	Ramesh et al. [56]	Zhao et al. [57]
Melting temperature [K]	468.6 ± 0.4	481.15 K	464.15 K
Melting enthalpy [kJ/mol]	27.47 ± 4	-	-

* The standard uncertainties u for melting temperature and fusion enthalpy are u(*T*) = 0.4 K and u(*H*_f_) = 4 kJ/mol.

**Table 2:** The solubility of chlorogenic acid in pure ethanol and water at atmospheric pressure.

Temp. * [K]	Solubility in Ethanol		Solubility in Water	
	ppm	Mole Fraction	ppm	Mole Fraction
298.15	3.18 × 10^5^ ± 1.12 × 10^3^	0.0571 ± 2.79 × 10^−4^	2.04 × 10^4^ ± 1.80 × 10^3^	0.0011 ± 9.53 × 10^−5^
303.15	3.37 × 10^5^ ± 1.01 × 10^4^	0.0619 ± 2.62 × 10^−3^	2.21 × 10^4^ ± 2.44 × 10^2^	0.0012 ± 1.29 × 10^−5^
308.15	3.83 × 10^5^ ± 5.67 × 10^3^	0.0748 ± 1.66 × 10^−3^	3.13 × 10^4^ ± 9.45 × 10^1^	0.0016 ± 5.10 × 10^−6^
313.15	3.88 × 10^5^ ± 3.65 × 10^1^	0.0762 ± 4.19 × 10^−5^	4.14 × 10^4^ ± 1.10 × 10^2^	0.0022 ± 6.03 × 10^−6^
318.15	4.30 × 10^5^ ± 1.95 × 10^4^	0.0894 ± 6.49 × 10^−3^	5.74 × 10^4^ ± 1.37 × 10^3^	0.0031 ± 7.77 × 10^−5^

* The standard uncertainties u for temperature and pressure are u(*T*) = 0.5 K.

**Table 3:** The solubility of chlorogenic acid in different mixed solvents at atmospheric pressure.

Temp. * [K]	Weight Fraction of Water	Solubility in Mixed Solvents	
		ppm	Mole Fraction
298.15	0.1807	3.51 × 10^5^ ± 9.24 × 10^2^	0.0485 ± 1.87 × 10^−4^
	0.3436	3.45 × 10^5^ ± 1.10 × 10^2^	0.0384 ± 1.80 × 10^−5^
	0.5393	2.69 × 10^5^ ± 1.43 × 10^4^	0.0231 ± 1.64 × 10^−3^
	0.7779	7.45 × 10^4^ ± 4.37 × 10^2^	0.0045 ± 2.86 × 10^−5^
308.15	0.1725	3.97 × 10^5^ ± 2.85 × 10^3^	0.0586 ± 6.57 × 10^−4^
	0.3235	4.20 × 10^5^ ± 4.52 × 10^3^	0.0521 ± 9.16 × 10^−4^
	0.4919	3.35 × 10^5^ ± 9.82 × 10^1^	0.0307 ± 1.31 × 10^−5^
	0.7281	1.48 × 10^5^ ± 8.88 × 10^2^	0.0098 ± 6.80 × 10^−5^
318.15	0.1648	4.63 × 10^5^ ± 6.04 × 10^3^	0.0753 ± 1.69 × 10^−3^
	0.3109	4.78 × 10^5^ ± 8.98 × 10^3^	0.0648 ± 2.18 × 10^−3^
	0.4496	4.60 × 10^5^ ± 7.72 × 10^2^	0.0520 ± 1.53 × 10^−4^
	0.6471	2.92 × 10^5^ ± 6.08 × 10^2^	0.0228 ± 6.55 × 10^−5^

* The standard uncertainties u for temperature and pressure are u(*T*) = 0.5 K.

**Table 4:** Information on chemical materials and their supplier.

Chemical Material	CAS Number	Structure	Molecular Weight [g/mol]	Source	Purity [%]
Chlorogenic acid (C_16_H_18_O_9_)	327-97-9	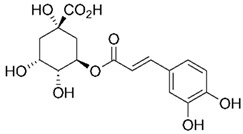	354.311	ALADDIN, China	98
Ethyl alcohol (C_2_H_6_O)	64-17-5	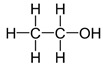	46.069	DAEJUNG, Republic of Korea	Anhydrous, 99.9

## Data Availability

The raw data of this article will be made available by the authors on request.
